# Physiological Measurements of Stress Preceding Incidents of Challenging Behavior in People With Severe to Profound Intellectual Disabilities: Longitudinal Study Protocol of Single-Case Studies

**DOI:** 10.2196/24911

**Published:** 2021-07-21

**Authors:** Rianne Simons, Renske Koordeman, Peter de Looff, Roy Otten

**Affiliations:** 1 Department of Research and Development Pluryn Nijmegen Netherlands; 2 Behavioural Science Institute Radboud University Nijmegen Netherlands; 3 Specialized and Forensic Care Wier (SGLVG Treatment Center) Den Dolder Netherlands; 4 Specialized and Forensic Care De Borg National Expertise Center Den Dolder Netherlands; 5 Research and Education Advancing Children's Health Institute Department of Psychology Arizona State University Tempe, AZ United States

**Keywords:** challenging behavior, electrodermal activity, heart rate, intellectual disability, single-case research, stress

## Abstract

**Background:**

Clients with severe to profound intellectual disabilities (SPID) and challenging behavior (CB) and the professional caregivers that support them are vulnerable to high stress levels, which negatively impact their well-being and the quality of care. CB is thought to result from an increase in the intensity and frequency of clients’ stress experiences. In turn, staff members experience stress in dealing with this behavior, and stressed staff members might behave in ways that increase clients’ stress levels, contributing to the origin and maintenance of CB. Research into these dyadic interactions between clients and staff is scarce for people with SPID, especially in real-life situations. The barriers of studying stress in this population include clients’ difficulties in communicating stress experiences and the lack of an objective continuous measure of stress.

**Objective:**

This paper presents a protocol for studying patterns of physiological stress in 15 client-caregiver dyads in the 30 minutes preceding incidents of CB compared to control periods without CB and the interplay between the stress levels of clients and professional caregivers.

**Methods:**

We will conduct 15 single-case studies to assess patterns of physiological stress in dyads of clients with SPID and professional caregivers prior to CB in several Dutch residential institutes. Client-caregiver dyads will wear the Empatica E4 wristband for 20 sessions of 3 to 8 hours without interruptions of daily routines while caregivers report clients’ CB. The physiological measures obtained will be electrodermal activity (microsiemens) and heart rate (beats per minute). A multilevel model with repeated measures at the incident level nested within the person level will be applied, employing separate models for electrodermal activity and heart rate to compare stress levels in the 30 minutes prior to incidents with control epochs. Covariates in the models include movement, temperature, and gender. In addition, cross-recurrence quantification analyses will be performed to study the synchronization between the stress levels of clients and professional caregivers.

**Results:**

The Ethics Committee of the Radboud University (NL-number: NL71683.091.19) approved the study on February 12, 2020. In total, 15 organizations have declared their commitment to participate in the study. The first result is expected in the spring of 2022.

**Conclusions:**

Study results will demonstrate whether changes in patterns of electrodermal activity and heart rate are apparent in the 30 minutes preceding an incident of CB compared to baseline levels when the client does not engage in CB. The synchronization between caregivers’ and clients’ physiological stress levels will be explored with cross-recurrence quantification analyses. Insights into the physiological stress levels of clients and caregivers may contribute to a reduction of CB and an improvement of both clients’ and caregivers’ safety and well-being.

**International Registered Report Identifier (IRRID):**

DERR1-10.2196/24911

## Introduction

### Background

According to the Dutch Healthcare Inspectorate, approximately 20% of the 30,000 people with intellectual disabilities (ID) living in Dutch residential institutes show severe and enduring challenging behavior (CB) [1]. CB most often includes aggression or self-injury [1] and can severely diminish clients’ quality of life [2]. The prevalence of CB increases with the severity of ID, resulting in a vulnerable group of clients with severe to profound ID (SPID) and CB [1,3]. Clients with SPID, especially in the case of CB, depend to a great extent on the support of professional caregivers in residential institutes for their physical and emotional well-being. It is well-established that CB can be stressful for caregivers, affecting caregivers’ well-being and the quality of support they provide [4]. Caregivers have reported that they are often surprised by clients’ CB and perceive the mere possibility of CB occurrence as highly stressful [5], which in turn may affect the stress experienced by clients. In addition, clients with SPID and CB perceive stress in a more frequent, intense, and sustained manner compared to the general population but lack sufficient coping skills to manage stress. Associations between stress and the origin and maintenance of CB have been reported, and CB is often a maladaptive response to stress [2]. This study addresses stress in clients with SPID and professional caregivers prior to incidents of CB.

### The Importance of Professional Caregivers’ Stress

Caregivers of clients with CB report more severe stress levels than other professionals serving clients with ID [6]. In several studies, caregivers rated clients’ CB as a highly stressful aspect of their work, and associations between the exposure to CB and caregivers’ stress have been repeatedly shown [7,8]. These associations may be moderated or mediated by caregivers’ attributions, coping styles, beliefs, self-efficacy, and training [7,8]. Specifically, Bromley and Emerson [9] reported that stress when supporting clients with CB is related to “the ‘daily grind’ of caring, their difficulty in understanding the person’s behavior, the unpredictability of the behavior, and the apparent absence of an effective way forward”. Several organizational variables (eg, workload, job variety, and support) have been identified as stressors as well [8,10]. CB and stress have a clear impact on caregivers’ psychological well-being and are associated with sickness, absenteeism, and staff turnover [10,11]. Importantly, stress impacts the quality of care [12]. The quality of care benefits from high-quality interactions between caregivers and clients [13], and inadequate caregiver-client interactions contribute to the origin and maintenance of CB [14]. Stress negatively affects the quality and quantity of these interactions and the caregiver’s ability to deal with CB effectively [4,7]. Specifically, stressed caregivers interact less often with clients, engage in more negative and less positive interactions, and are more likely to behave in ways that contribute to the origin and maintenance of CB [7,14].

### Understanding Stress in Clients With SPID and CB

CB may be a maladaptive response to perceived stress, and research has shown an association between clients’ stress and CB [2]. Janssen, Schuengel, and Stolk [2] applied an explanatory model based on theories about stress and attachment to the development of CB among clients with SPID. According to this stress-attachment model, the combination of stress and insecure attachment puts people with SPID at risk for CB. Specifically, people with SPID perceive stress in a more frequent, intense, and sustained manner when compared to the general population, but often lack appropriate coping skills to deal with stressors [2]. To deal with stressors without sufficient coping skills, people fall back on the support of an attachment figure (ie, someone to whom one is securely attached) [15]. Access to typical attachment figures (ie, parents, close friends, and mentors) is limited in residential institutes [16]. Therefore, secure attachment to professional caregivers is important for clients to receive emotional support in stressful situations. Indeed, clients who show more secure attachment behavior towards caregivers show less CB [15]. Clients who do not experience high-quality interactions with caregivers are at risk of developing insecure attachment relationships, and an accumulation of stress might cause CB [17]. Limited cognitive skills have been identified as precursors of insecure attachment, increasing the risk for CB following stress in people with SPID [2].

### Physiological Indicators of Stress

One of the difficulties in studying stress in clients with SPID and CB is the communicative shortcomings associated with clients’ ability and caregivers’ difficulties interpreting clients’ communicative signals [2]. Although caregivers try to anticipate CB by relying on the mixed and difficult-to-interpret signals from clients that might indicate stress, caregivers lack insight into clients’ stress experiences and report being surprised by CB [5]. Further, most research on caregiver stress relies on a range of questionnaires, measuring subjective experiences with variations based on, for example, caregivers’ age and experiences. The results from these studies are hard to interpret due to methodological differences in measuring stress and the lack of an objective, continuous measure of stress [10]. Since psychologically stressful events change people’s physiology, studying physiological stress increases insight into patterns of stress that precede incidents of CB [2]. In addition, physiological measures of caregiver stress are significantly associated with burnout symptoms over time [11]. Both physical and emotional stress result in physiological responses characterized by the dual innervation of the autonomous nervous system [18]. During stressful events, increased activity of the sympathetic nervous system and decreased parasympathetic nervous system activity is typically observed, resulting in increased heart rate, electrodermal activity, and blood pressure [19]. However, more complex relations between the innervation of both branches and aggression have been observed as well [20]. Although physiological measures of stress have been studied repeatedly, most studies are conducted in experimental settings. The development of wearable wireless devices has increased the possibilities of measuring physiological indicators of stress nonintrusively and continuously in real-life settings [5]. Electrodermal activity (EDA) and heart rate (HR) are reliable physiological measures of stress that can be monitored with these wearables [21]. Some naturalistic studies have been performed to assess physiological stress using wearables in real-life settings. Rises in EDA [22-24] and HR [22] have been reported in the 30 minutes prior to aggression in psychiatric patients. Physiological stress and motion measures can be used to predict aggression to others in the upcoming minutes in people with autism spectrum disorder (ASD) [25]. In an earlier study, Noordzij, Scholten, and Laroy-Noordzij [5] have shown the possibilities of measuring EDA and HR in clients with SPID and the caregivers that support them during incidents of CB. In inpatient forensic mental health services, caregivers reflected on the utility of technological devices to provide early warnings for impending aggression [26].

### Our Study

**Figure 1:**
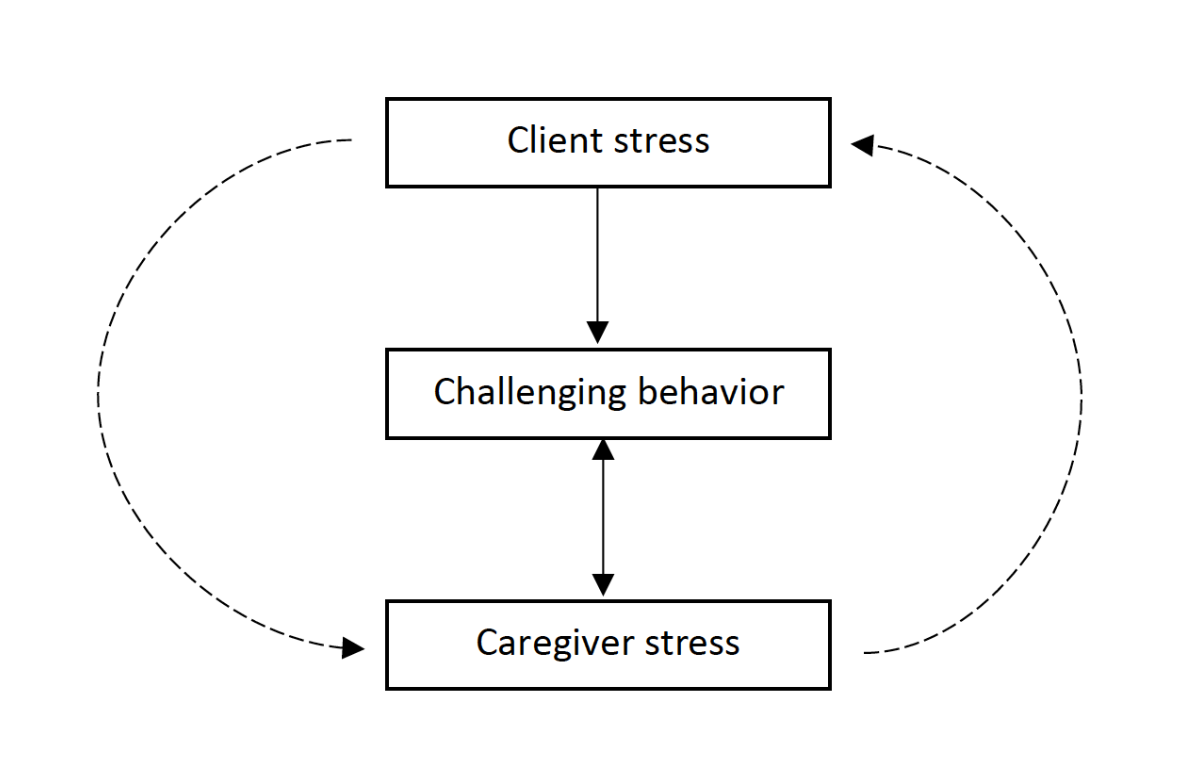
A reciprocal model of stress and challenging behavior in caregivers and people with severe to profound intellectual disability.

The literature has shown the predictive value of physiological measures of stress for upcoming aggression in psychiatric patients and people with ASD. Although the possibilities of using wearables to study physiological stress in clients with SPID and CB have been shown, not much is known about patterns of physiological stress prior to CB in clients with SPID or in the caregivers that support them. Since the existing literature shows the well-being of caregivers is affected by stressors associated with clients’ CB, and the relationship with caregivers is important for clients to cope with stress and prevent CB, studying the reciprocity between clients’ and caregivers’ physiological stress is an eminent step in further increasing insights into patterns of stress and the origin of CB. Therefore, we hypothesize a reciprocal model in this study of stress and CB (Figure 1). The model suggests that: (1) client stress is associated with CB [2,15,17], (2) caregiver stress contributes to CB [4,7,14], (3) CB contributes to caregiver stress [6-9], and (4) caregiver stress impacts client stress and vice versa.

This naturalistic study explores patterns of physiological stress (ie, EDA and HR) in clients with SPID and professional caregivers prior to CB in Dutch residential institutes and the synchronization between client and caregiver patterns of stress. We address the following research questions:

1. Do patterns of physiological stress in clients with SPID and professional caregivers in the 30 minutes preceding an incident of CB differ from control periods of 30 minutes without CB?
2. Do the physiological stress levels of caregivers impact clients’ physiological stress levels and vice versa?

We hypothesize that a significant rise in EDA and HR is apparent in both clients and caregivers in the 30 minutes preceding an incident of CB compared to baseline levels when the client does not engage in CB. In addition, we hypothesize physiological synchrony—a mutual change in autonomic nervous system activity—between caregivers and people with SPID and CB. Physiological synchrony has been shown in some interaction partners, including parent-infant dyads [27,28], strangers [29], and romantic couples [30]. However, to our knowledge, physiological synchronization has not been studied before in dyads of professional caregivers and clients with SPID and CB.

## Methods

### Study Design

A longitudinal design will be performed in 15 single-case studies in which one case is a dyad of one unique client and one unique professional caregiver. More so than randomized controlled trials, multiple single case studies offer opportunities to study underlying processes and perform elaborate analyses on the individual and the environmental level [31]. In addition, people generally respond differently to stressful events and interpret different situations as stressful due to age, gender, experiences, and so on [32], underpinning the usefulness of single-case designs. The study will be performed in Dutch residential care organizations for people with ID. The organizations are part of a knowledge platform (in Dutch: “Kennisplatform EVB”) for sharing and developing knowledge regarding people with SPID and CB. This study was approved by the Faculty Ethics Committee of the Radboud University (NL-number: NL71683.091.19) February 12, 2020. Informed consent will be obtained from all caregivers and parent(s) or legal representative(s) of clients.

### Sample Size

This study included 15 dyads of a client and a professional caregiver. Due to the exploratory nature of the study, the sample size is not calculated. The sample size of 15 was determined based on feasibility due to the time investment necessary per case and the resources available to conduct this study. Single-case designs have additional sample size factors involved in determining power and accuracy besides the number of participants, including the number of observations [33]. In this study, we use several measurements for each dyad, increasing the power with a relatively small sample size.

### Eligibility

Clients will be eligible to enter the study if they meet the following inclusion criteria: (1) adults between 18 and 65 years of age, (2) anticipated to engage in CB regularly (ie, multiple times per week), (3) expected to reside in a designated institute for the upcoming 3 months, and (4) an IQ of 40 or below. Recruited caregivers (1) work a minimum of two days a week with the client and (2) are expected to work with the client for the upcoming 3 months. Clients for whom caregivers, behavioral experts, parents, or legal representatives expect to experience excessive distress from wearing the wearable, or will most likely damage the wearable, are excluded.

### Recruitment

Participants will be recruited during a symposium concerning clients with SPID and CB and through invitation letters among the 28 organizations that are part of the knowledge platform. First, the researcher informs interested caregivers about participation in the study via telephone or face-to-face discussions. Second, caregivers will indicate which client is eligible to participate in the study. Third, caregivers will contact clients’ parent(s) or legal representative(s) to inform them about the study with an information letter and request for consent to participate. Informed consent will be obtained from all caregivers and legal representative(s) of clients.

### Study Procedure

Clients and caregivers will wear the wearable during 20 sessions of 3 to 8 hours. The sessions do not interfere with daily routines and activities. The caregiver will attach the wearable to their own and their client’s wrist. Caregivers will follow a protocol developed specifically for clients with SPID and CB to attach the wearable to the client’s wrist because the attachment of the wearable may induce stress for both caregivers and clients due to the deviation from the daily routine [5] (see Multimedia Appendix 1). The protocol is personalized per dyad. To minimize the influence of the wearable on stress and behavior, clients and caregivers will wear the wearable prior to the study to familiarize themselves with it. When the wearable no longer induces stress to the client and the caregiver, we will start collecting measurements. The data are saved in the memory of the device and exported into a secured server via USB.

### Measures

#### Physiological Stress

Physiological measures of stress are obtained with the Empatica E4 wristband, a wearable wireless device that measures EDA, blood volume pulse (from which interbeat intervals and HR are derived), body temperature, and movements. This wearable provides advanced data quality compared to other available wearables, including artifact removal technique [34]. Validation studies show promising results [35,36].

HR is determined with a photoplethysmography (PPG) sensor, acquired at a 64 Hz sampling frequency. The amount of light through the skin reflected on the PPG sensor reflects the blood volume changes in the vessels with each heartbeat. HR (beats per minute) is derived from the blood volume pulse. EDA (microsiemens) is acquired at a 4 Hz sampling frequency using two electrodes through which a continuous current flows. When one’s sympathetic arousal level increases during stressful events, more sweat is produced, which increases EDA, whereas decreased sympathetic arousal results in a decrease in EDA. The extracted parameters are skin conductance level (SCL) and the number of peaks per minute (PPM). Measures of HR and EDA are corrected for temperature (degrees Celsius) and movement (measured with a 3-axis accelerometer) [34].

#### Challenging Behavior

For each incident that occurs during the sessions that the E4 wristband is worn, caregivers will report the following: date, time, type and severity of the incident, a description of the incident, whether the incident was expected (yes or no), and location. The incident registration form is based on the Modified Overt Aggression Scale [37]. The incidents are classified as either verbal aggression, aggression against property, auto aggression (ie, self-injury), or physical aggression. The severity of an incident is scored on a scale from 0 (light) to 4 (severe). Examples per type of incident are provided per severity score and personalized per client. Caregivers fill in the incident registration form on paper immediately after the incident has been averted.

#### Client Mood

Caregivers indicate the client’s mood every 15 minutes with green, yellow, orange, and red cubes. The definition of the colors is determined per client with caregivers and behavioral experts by associating each color to specific behaviors. Client mood scales will be based on a daily questionnaire reported in the electronic client record by some organizations. In addition, every organization makes use of a “signaling plan,” which includes 4 phases of behavior ranging from 0 (relaxed) to 4 (extremely stressed). The behaviors in these plans are translated into the 4 colors used to indicate mood in this study. To ensure feasibility, caregivers estimate the client’s mood throughout the day but only report the client’s mood on paper at the beginning of each shift and when the client’s mood changes.

#### Self-Reported Stress

Self-reported caregiver stress in the past month is measured prior to and after participation in the study. Caregivers fill in the 10-items Perceived Stress Scale (PSS-10) [38], which measures the degree to which situations are appraised as stressful on a 5-point Likert scale from 0 (never) to 4 (very often). A sum score between 0 and 40 gives an indication of perceived stress in the last month. Psychometric qualities are good [39]. The Dutch translation of the PSS-10 will be used.

In addition, self-reported caregiver stress in the 30 minutes prior to an incident of CB is measured on a scale from 1 (totally relaxed) to 10 (totally stressed out).

#### Demographics

The following client characteristics will be collected: sex, age, IQ, history of incidents, diagnoses, and medication. The following caregiver characteristics will be collected: sex, age, education, and work experience.

### Statistical Analyses

Descriptive statistics regarding incidents of CB and stress levels will be described in relation to demographic data. As measuring physiological stress in real-life situations results in artifacts (ie, disturbances in the signal due to excessive movements), the data will be visually inspected, and impossible values removed. Subsequently, data will be corrected automatically with a proven method described by Taylor and colleagues [40]. Statistical analyses will be performed in R-4.0.4.

EDA and HR of clients and caregivers in the 30 minutes prior to an incident of CB will be compared with 30 minutes of control periods in which the client did not engage in CB. A multilevel model with repeated measures (level 1) at the incident level (level 2) nested within the person level (level 3) will be used. For the repeated measures (level 1), the 30-minute sessions are divided into epochs of 5 minutes. Separate models for PPM, SCL, and HR will be considered. Covariates in the models include movement, temperature, and gender.

Recurrence Plots (RPs) of the 30 minutes prior to an incident of CB will be made. Cross-Recurrence Quantification Analysis (CRQA) will be performed to determine if patterns of stress in the time series of caregivers are recurring in the time series of clients, and vice versa, in a moment prior, simultaneously, or later in the time series [41]. The Recurrence Rate (RR), a ratio of the number of recurrent points over the total possible number of recurrent points, will be calculated. The RR indicates how often a point in one time series recurs in another time series. Determinism, which is the number of recurrent points that form a diagonal line in an RP relative to the total number of recurrent points, will be calculated. Determinism represents the recurrence of patterns over time, and a high determinism indicates the synchronization of patterns of physiological stress between the caregiver and the client. A diagonal Cross-Recurrence Profile provides insights into who is leading in the pattern of physiological stress prior to an incident. In other words, is the recurring pattern of stress first noticeable in a client and subsequently in the caregiver, or vice versa? CRQA is performed using the R package “crqa” [41].

## Results

In total, 15 organizations have declared their commitment to participate in the study. Research findings will be disseminated through peer-reviewed journals, professional networks, conferences, and the website of the knowledge platform concerning people with SPID and CB. The first results are expected in the spring of 2022.

## Discussion

### Conclusions

This protocol study describes a series of single-case studies assessing stress patterns in professional caregivers and clients with SPID prior to incidents of CB in Dutch residential institutes and the synchronization or desynchronization between these patterns. We hypothesize that a significant rise in EDA and HR is apparent in clients and caregivers in the 30 minutes preceding an incident of CB compared to control periods without CB. In addition, we expect a reciprocal influence of caregivers’ and clients’ stress levels, meaning an increase in caregiver stress is followed by the rise in client stress and vice versa.

One of the study's strengths is that stress is measured in a real-life setting without interfering with daily routines. While measuring physiological stress in real-life settings results in artifacts, the models in this study will be corrected for movement and temperature. A visual and an automatic correction of artifacts will be performed as well [40]. In an earlier study [22], a considerable number of artifacts, besides movement and temperature, were found in the data related to the tightness of the wearable on the wrist, which will be addressed when instructing participants. Besides movement and temperature, gender is included as a covariate in the models. Meta-analyses have shown that the associations between physiological measurements and behavior are similar for men and women, but the strength of the associations varies [42-44].

Another strength of this study is that interpersonal variations are accounted for by comparing stress of the same subject in periods prior to CB with control periods without CB. This comparison is important as people have different baseline values of EDA and HR [22]. In addition, the stress of both client and caregiver is assessed in the same time frame. This enables us to study the reciprocity between client and caregivers’ stress, which is important as client-caregiver interactions impact the development of stress and CB and are vital to the quality of support caregivers can provide [2,13].

A limitation of the study is that the available resources (eg, time and money) restrict our ability to document fully detailed contextual factors that may affect the outcome. For instance, specific reasons why a caregiver or client is stressed prior to CB remain unaddressed, and variations in physiological stress patterns prior to CB due to other causes of stress that were not assessed remain unknown.

Finally, heart rate variability (HRV) is commonly used as a physiological measure of stress; it is a more sensitive measure for stress when compared to HR, which will be used in this study [21]. However, the amount of participants’ movement in real-life settings significantly interferes with the accurate registration of HRV with a PPG sensor [22,45,46]. For this reason, HR will be used as a physiological measure of stress rather than HRV.

### Implications for Practice

Stress is common in clients with SPID and CB and the caregivers that support them, and it is often a precursor of CB. Measuring physiological stress is important due to the communicative difficulties associated with the severity of ID and CB, especially in clients with SPID [2], and the reported difficulties of caregivers in interpreting clients’’ cues of stress [5]. Measuring physiological stress provides insights into the real-life emotions of those who have difficulties expressing emotions to their caregivers, and it offers a first step in increasing insights into the processes of stress that precede incidents of CB. Eventually, these insights can assist caregivers in recognizing CB by informing them about changes in client stress levels in order to anticipate clients’ CB. In addition, caregivers are informed about changes in their stress levels to enable effective stress coping. Insights into the stress of clients and caregivers will contribute to their safety and well-being.

## Appendix

Multimedia Appendix 1 Protocol to attach the wearable to the wrist of a person with severe to profound intellectual disability and challenging behavior [<xref ref-type="bibr" rid="ref5">5</xref>].

## Abbreviations

ASD: autism spectrum disorder
CB: challenging behavior
CRQA: cross-recurrence quantification analysis
EDA: electrodermal activity
HR: heart rate
HRV: heart rate variability
ID: intellectual disability
PPG: photoplethysmography
PPM: peaks per minute
PSS-10: 10-items perceived stress scale
RP: recurrence plot
RR: recurrence rate
SCL: skin conductance level
SPID: severe to profound intellectual disability
