# Supplementary material for: Physiological Measurements of Stress Preceding Incidents of Challenging Behavior in People With Severe to Profound Intellectual Disabilities: Longitudinal Study Protocol of Single-Case Studies
Source: JMIR Res Protoc. 2021 Jul 21;10(7):e24911. doi: 10.2196/24911 (PMC8339975; doi:10.2196/24911)
Supplement: Multimedia Appendix 1 [file resprot_v10i7e24911_app1.pdf]

Wait 5 seconds following each instruction

1

Use a pictogram to communicate in the daily program of the client

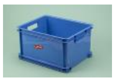

2

Take the crate with the materials to the client

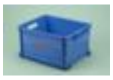

3

Show the crate with the materials tot he client

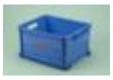

4

Say: “ Hi …, it is time to put on the wristband”

5

Put on your own wirstband (left pols) in front of the client (optionally, make use of doll)

6

Show wearable

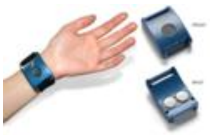

7

Show wearable + say: “extent your left arm and put on the wristband”

8

Show wearable + “extent your left arm” + grab client’s wrist and attach the wristband

9

Turn on the wearable (follow instructions to start a measurement)

10

Measurment has started
